# Simulation Studies on Centrifugal MQL-CCA Method of Applying Coolant during Internal Cylindrical Grinding Process

**DOI:** 10.3390/ma13112506

**Published:** 2020-05-31

**Authors:** Seweryn Kieraś, Marek Jakubowski, Krzysztof Nadolny

**Affiliations:** 1 Wartsila Poland Sp. z o.o., Łużycka 2, 81-537 Gdynia, Poland; seweryn.kieras@wartsila.com; 2 Department of Food Industry Processes and Facilities, Faculty of Mechanical Engineering, Koszalin University of Technology, Racławicka 15-17, 75-620 Koszalin, Poland; marek.jakubowski@tu.koszalin.pl; 3 Department of Production Engineering, Faculty of Mechanical Engineering, Koszalin University of Technology, Racławicka 15-17, 75-620 Koszalin, Poland

**Keywords:** CFD simulation, internal cylindrical grinding, MQL, CAG

## Abstract

This paper describes simulation studies regarding the application of the centrifugal minimum quantity lubrication (MQL) method simultaneously with the delivery of a compressed cooled air (CCA) stream in the internal cylindrical grinding process. The idea of a new hybrid cooling and lubrication method connecting centrifugal (through a grinding wheel) lubrication by MQL with a CCA stream is described. The methodology of computational fluid dynamics (CFD) simulation studies, as well as the results of numerical simulations, are presented in detail. The aim of the simulations was to determine the most favourable geometrical and kinematic parameters of the system in the context of air-oil aerosol and CCA flow, as well as heat exchange. In the simulation, the variables were the grinding arbor geometrical parameters, the angle of CCA supply line outlets, and the grinding wheel and workpiece peripheral speed. As a result of the simulation studies, the most favourable geometrical parameters were designated, determining the orientation of the ends of the two CCA supply line outlets before and after the grinding zone, the number of openings in the drilled-out grinding arbor, and the influence of the grinding speed on the parameters of the coolant flow and temperature of objects in the grinding zone. In addition, the results of simulation tests made it possible to visualise the velocity vectors of the two-phase coolant flow in a complex system of air-oil aerosol delivery centrifugally through an open structure of a very fast rotating porous layer (grinding wheel), with an additional supply of CCA using an external cold air gun (CAG).

## 1. Introduction

Internal cylindrical grinding processes are among the most demanding types of grinding processes, mainly due to their kinematics [1,2]. The greatest difficulty is the elongated contact zone between the grinding wheel and the workpiece material, caused by a small difference between the diameter of the wheel and the opening being ground. The difficult conditions of the described process make the proper cooling and lubrication of the grinding wheel active surface (GWAS) contact zone with the workpiece extremely important for obtaining high quality of the machined surface and for the repeatability of the process in the long term.

The mechanical energy entered into the grinding as a result of relative movement of the tool and the workpiece is largely converted into heat [1,2]. This leads to a significant increase in temperature in the grinding wheel/workpiece contact zone, caused by friction and deformation leading to chip formation and material removal. The elongated contact path between the components of the active surface of the grinding wheel and the ground surface makes the heat dissipation from the grinding zone one of the most important factors determining the effectiveness of the machining process. Excessive increase in temperature during the grinding process can lead to surface defects such as micro-cracks, grinding burns or unfavourable stresses on the surface layer. In addition, an increasing temperature in the grinding zone causes excessive wear of abrasive grains and bond. This leads to plasticisation of the vertices of active abrasive grains. It can also cause thermal wear of bond bridges and crushing of grains from the GWAS [1,2,3,4]. In extreme cases, a rapidly changing temperature gradient in the abrasive tool may cause excessive thermal stress, resulting in a drastic decrease in the strength of the entire tool and its tearing.

The long contact path is also conducive to the formation of cloggings on the GWAS made from chips of machined material, especially in the case of increased and high material removal rates. This is due to the difficult transport of grinding products out of the grinding zone in the intergranular space. Cloggings, apart from an obvious decrease in the grinding wheel’s cutting ability, increase the share of friction and influence the increase in temperature in the zone of contact with the machined surface [5,6,7,8,9,10,11,12,13,14].

An additional problem concerns the difficulty in delivering coolant to the grinding zone. The most commonly used flood method does not ensure uniform delivery of the coolant, the effectiveness of which decreases as the grinding wheel moves into the machined opening. Due to the small size of the grinding wheel, it is not possible to use more advanced coolant delivery techniques, e.g., pressure or shoe nozzles, as is the case with surface or external cylindrical grinding processes [5,6,7,8,9,10,11,12,13,14]. It follows from the above that the type, output and method of coolant delivery have an extremely important impact on the repeatability of the internal cylindrical grinding process, as well as on the quality of its results.

Taking into account the specific problems occurring in the internal cylindrical grinding process [1,2], several major issues have been focused on developing solutions to increase the efficiency of methods of cooling and lubrication of the machining zone, while reducing their negative impact on the environment:

- the possibilities of increasing the coolant’s penetration efficiency directly into the GWAS contact zone with the workpiece surface;
- the possibilities of reducing the number of coolants used in the grinding process;
- the elimination of the need to use coolants by looking for new media.

The analysis of known methods of delivery of coolants, lubricants and antiadhesive agents to the grinding zone made it possible to select centrifugal coolant delivery (through the grinding wheel) as being the most advantageous with respect to increasing the efficiency of reaching the grinding zone. Solutions of this type have been described for many years in the process of grinding with large-sized grinding wheels (surface grinding, external cylindrical and shape grinding) due to the relatively large space around the grinding wheel and easy access to it by special coolant delivery systems. Therefore, work has been undertaken to develop innovative methods of centrifugal coolant feeding in the case of using small-sized grinding wheels, such as those used in the internal cylindrical grinding processes, for which there are many more limitations in the implementation of centrifugal cooling. At the same time, these works took into account the possibility of minimising the amount of coolants delivered. In this context, the most frequent description in the literature is the minimum quantity lubrication (MQL) method, the possibilities of which have been analysed in relation to machining [15,16] as well as with respect to the grinding process in general [17,18,19,20,21,22,23,24,25,26,27,28]. Most often, researchers focus on surface grinding process [29,30,31,32], but the possibilities of the MQL method in the grinding of hard-to-cut materials have also been considered [33,34,35,36]. Other known methods of minimising coolant expenditure include: Minimum quantity cooling (MQC) [37,38,39], minimum quantity cooling lubrication (MQCL) [40,41,42,43,44,45,46,47], cold air minimum quantity lubrication (CAMQL) [24,48,49], and cold air and oil mist CAOM [50,51,52,53,54].

The integration of the MQL method and the delivery of a compressed cooled air stream known as cold air and oil mist seems particularly advantageous. This solution was presented in works [50,51], where a system consisting of a CAG nozzle and a MQL nozzle was used. Based on the results of the research, it was shown that the application of the CAOM hybrid method of cooling and lubrication makes it possible to prevent the occurrence of changes in the structure of the machined surface layer in the form of burns. In addition, a decrease in the grinding force values in relation to the dry machining method was noted. However, due to the accumulation of thermal energy in the workpiece, removal of the workpiece material with an increased depth (above *a_e_* = 15 µm) resulted in the formation of grinding burns [50,51].

At the same time, Yui and Terashima [54] showed a positive influence of a small amount (output of up to 8.6 cm^3^/h) of vegetable oil additive on the conditions of the grinding process with the help of compressed cooled air. The use of vegetable oils made it possible to achieve grinding force values as much as 10% lower, with comparable values of surface irregularities in relation to the results obtained with the use of conventional oil cooling. The value of grinding ratio *G* using the CAG nozzle cooling method and with the addition of vegetable oils increased almost twofold in comparison to the process carried out with the flood method with oil [54].

Additionally, Stachurski et al. [53] used a hybrid CAOM method, which consists of delivering air-oil aerosol and compressed cooled air into the grinding zone during the sharpening process of worm milling cutters. The lubricant in the form of an air-oil aerosol was delivered with an output of 50 mL/h onto the active surface of the rotating wheel, so it was delivered directly to the grinding zone. Cooled to −5 °C, compressed air was mainly used as a cooling medium and wheel clogging was minimised by removing chips and grinding products that remained after leaving the grinding zone from the active surface. The described tests were carried out by comparing the results of grinding the abrasive surface of worm cutters using the flooding method, the CAOM method, and the MQL method, as well as delivery-only CCA. The obtained results showed that the application of the CAOM method made it possible to prevent the occurrence of undesirable changes in the structure of the workpiece surface layer in the form of thermal defects. Sharpening of the worm milling cutters with the use of CAOM method in the range of applied grinding parameters did not cause significant changes in the microhardness of the surface layer in comparison with the flood method. The results obtained for the MQL and CCA methods used separately indicate a significant, unacceptable decrease in the microhardness of the cutter rake surface pick after grinding [53].

The advantages of the CAOM method described in works [50,51,52,53,54] consist primarily of effective support of the cooling function of MQL method without increasing the coolant expenditure, only by cooling the previously compressed atmospheric air in the vortex tube and directing it to the grinding zone. As a result, literature sources state that the application of the CAOM method in grinding processes prevents the occurrence of workpiece surface thermal defects, makes it possible to reduce the grinding force and increase the grinding ratio *G*.

The listed advantages of the CAOM method were the reason for conducting the research aimed at implementing CAOM method in internal cylindrical grinding process (until now, such a method of cooling and lubrication has been described only in the processes of surface grinding, external cylindrical and shape grinding). As a consequence of the work carried out, a centrifugal MQL-CCA method has been developed. This new method is a merger of centrifugal air-oil aerosol delivery through the grinding wheel with the additional delivery of a CCA stream [55].

This paper presents the characteristics of the centrifugal MQL-CCA method integrating centrifugal lubrication with a minimum quantity of lubricant and cooling with a CCA stream—Section 2. Then, the methodology (Section 3.1), mathematical model (Section 3.2), and the results and analysis (Section 3.3) of the computational fluid dynamics simulation studies are described. Section 4 presents insightful conclusions on the parameters analysed in the simulation experiments.

## 2. Characteristic of the Centrifugal MQL-CCA Method of Applying Coolant during Internal Cylindrical Grinding Process

**Figure 1:**
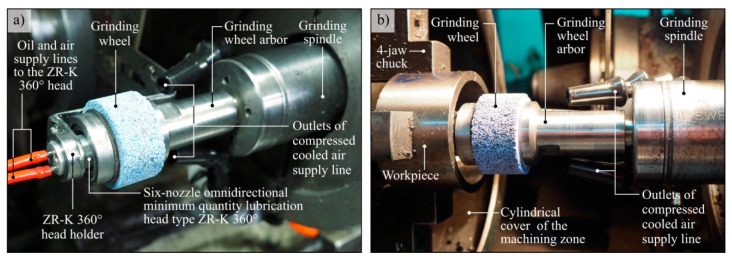
View of centrifugal MQL-CCA method components (**a**) installed in the grinding zone (**b**).

When configuring the components of the cooling system, a CAG nozzle and an air-oil aerosol centrifugal supply system were used (Figure 1), consisting of the following components:

- six-nozzle omnidirectional minimum quantity lubrication head type ZR-K 360°;
- MQL head supply system with compressed air and oil from the workpiece spindle side;
- special design grinding wheel arbor;
- system for fixing the ZR-K 360° head stationary inside the rotating grinding wheel arbor;
- special ceramic grinding wheel with dimensions 40 mm × 20 mm × 26 mm adapted to work with hollow grinding arbor;
- as a lubricant, an oil called Cimtech^®^ MQL from CIMCOOL^®^ Fluid Technology, part of Milacron LLC, was used.

In the immediate surroundings of the machining zone, a CAG nozzle of the type Vortec 610 was placed. This nozzle was equipped with a double-jet flexible supply line. One outlet of the supply line was directed in front of the grinding zone for cooling, while the other outlet was placed directly behind the grinding wheel’s contact zone with the workpiece to support the blowing of machining products. The outlets of the CAG nozzle supply line had openings with a diameter of 6.3 mm. The pressure of the air feeding the nozzle was 0.6 MPa, which made it possible to reduce the temperature of air at the outlet to approximately −5 °C. The total CAG flow rate for such CAG configuration was *Q_CCA_* = 49.8 dm^3^/min (0.00083 m^3^/s).

## 3. Simulation Tests of Coolant Flow and Heat Transfer in the Grinding Zone

The aim of the simulation studies was to determine the most favourable geometrical and kinematic parameters of the system in question in terms of the flow of cooling and lubricating media (air-oil aerosol and CCA) and heat exchange. The simulation was carried out for a total of 27 system variants, varying:

- the geometry of the grinding arbor (three variants of arrangement and number of channels on its circumference);
- the angle of inclination of the outlets of the CCA supply line to the grinding zone (three angle values);
- the peripheral speed of the grinding wheel *v_s_* and workpiece *v_w_* (three values of *v_s_* and *v_w_* speed).

In each variant of the simulation tests, the influence of the input variables on the direction of flow, speed and temperature of coolants, and grinding wheel and workpiece temperature was determined. The results of the different simulation variants made it possible to choose the most advantageous configuration of the tested system, without the necessity of carrying out analogous, real comparative tests. The obtained results of the simulation experiments were used to develop mathematical models of the research object (MMRO) describing the influence of input variables on selected result parameters of the analysed process.

### 3.1. Methodology of Simulation Tests

During the simulation tests, the following input values related to the model geometry were changed:

- number of channels in grinding arbor for air-oil aerosol centrifugal delivery: *n_channels_* = 3, 6, 9;
- the diameter of a single channel in the grinding arbor for air-oil aerosol delivery depended on their number in such a way that the total area of the channels cross-sectional area is constant: *d_channels_* = 5.00 mm (for *n_channels_* = 9); 6.12 mm (for *n_channels_* = 6); 8.66 mm (for *n_channels_* = 3);
- alignment angle in the *z* axis of the CCA supply line outlets: *α*_1_ = *α*_2_ = 10.0°; 12.5°; 15.0°;

and the kinematic parameters of the simulated process:

- peripheral speed of grinding wheel: *v_s_* = 40 m/s; 50 m/s; 60 m/s;
- peripheral speed of the workpiece (speed ratio *q* = *v_s_*/*v_w_* was taken as constant: *q* = 60): *v_w_* = 0.67 m/s, 0.83 m/s, 1.00 m/s.

In the simulation studies, a total of 27 numerical experiments were assumed to be carried out. Every point of the experimental plan was related to a different combination of input variable values. The output variables from the simulation process were: direction and speed of the air-oil aerosol flow, direction and speed of the CCA flow, and the temperature of these two streams. The results of the simulation studies were the temperature distribution in the volume of the grinding wheel and the workpiece. The flow rate of the air-oil aerosol and CCA and other geometrical parameters of the analysed system remained constant. The aim of the study was to determine the most advantageous variant of the combination of input values of the simulation, making it possible to obtain the highest flow velocity of the cooling lubricants and the lowest temperature of the grinding wheel and the workpiece. Selected physical and mechanical properties of the workpiece material (100Cr6 steel) are given in Table 1.

**Figure 2:**
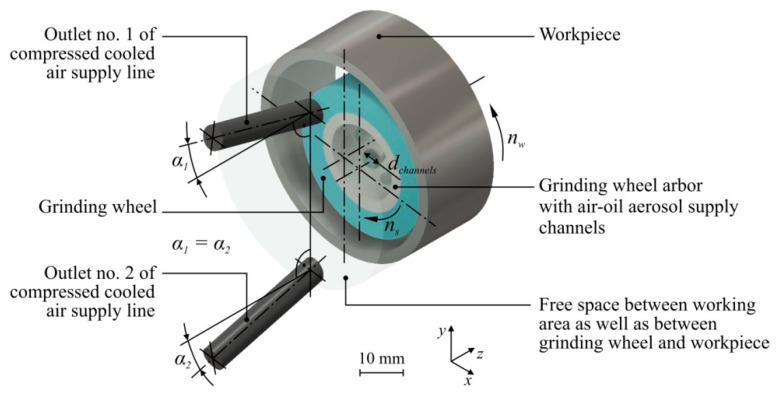
Geometrical model for simulation tests with main kinematic and geometric parameters: *α*_1_ and *α*_2_—alignment angle of compressed cooled air supply line outlet no. 1 and 2; *d_channels_*—grinding arbor channels diameter; *n_s_*—grinding wheel rotational speed; *n_w_*—workpiece rotational speed.

Figure 2 presents an axonometric view of the solid model developed for numerical simulation. The most important geometric and kinematic parameters of the model are also marked.

**Figure 3:**
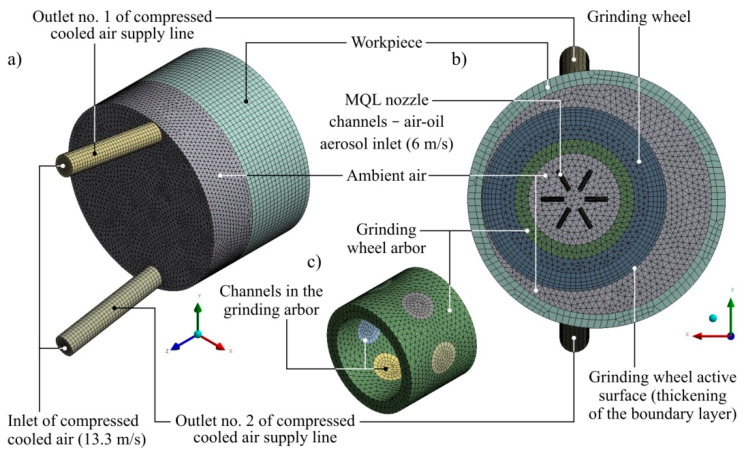
View of the simulation model solids after discretisation: (**a**) isometric view of all elements of the model; (**b**) view of all elements of the model in the *xy* plane; (**c**) isometric view of the grinding arbor model.

The simulation was conducted with Ansys CFX 18.1 software. In the simulation studies, the Finite Volume Method was used to solve systems of differential equations by dividing (discretisation) the fluid domain and performing calculations for the volume of mesh near nodes of the created elements. The view of individual solids of the considered system after discretisation is given in Figure 3.

The simulation was performed as a steady-state case. The number of iterations was set as 1000. The convergence criteria for each simulation case were residual of Root Mean Square (RMS) type with residua target set to 1 × 10^−4^. Each case achieved convergence after about 900 iterations.

The presented case was simulated like a turbulent flow. The two-dimensional k-ω SST model was used. The values of empirical coefficients of the model were set as defaults. Additionally, the blending functions were not considered at this stage of simulation. Model coefficients and blending functions are important in cases of flow with a high degree of tribalisation along with additional elements appearing in the analysis, such as the free surface, like in, among others, D’Ippolito et al. [56]. In the case under consideration, the simulation results reflect the actual state occurring on the surface of the grinding zone. Additional settings for the turbulence model were not required.

Various types of elements and their sizes were used to generate a discrete finite element mesh. Lumps of regular solids (workpiece geometry) and fluids bounded by a cylindrical wall (geometry of the CCA supply line outlets, the channels around the air-oil aerosol supply nozzle, and the channels in the grinding arbor) were divided into cubic discrete elements (hexahedrons). The use of cubic elements made it possible to discredit solids with less complicated geometries with a relatively small number of elements and knots (in relation to the mesh composed of quadriceps), which limited the duration of the simulation calculations. The remaining solids with less complex geometries, structures and predicted flow characteristics (geometry of the grinding wheel, grinding wheel and ambient air geometry) were divided into tetrahedrons, which are more adaptable to complex geometries, but which, however, may result in a greater demand for the calculation power of the workstation. Table 2, Table 3, Table 4 and Table 5 present a complete list of the simulation parameters.

Simulation studies were conducted according to the planned experiment. A complete three-level plan with three variables *v_s_*, *n_channels_*, *α* (with *α* = *α*_1_ = *α*_2_) was used. Both the planning of experiments as well as statistical analysis of the obtained simulation results were carried out with the use of specialist software Experiment Planner 1.1, which enables planning of exploratory and proper experiments as well as identification and analysis of the mathematical model of the research object.

### 3.2. Mathematical Model

The flow of a fluid is described by the conservation of mass (continuity) equation and the conservation of momentum equation, also known as the Navier–Stokes equations.

Equations for the conservation of momentum are derived by applying Newton’s law of motion to a small volume of fluid. Newton’s law states that the rate of change of momentum for a volume of fluid is equal to the sum of all external forces acting on that volume. The equation of velocity balance (1) takes the following form:

(1) $$\rho \frac{\partial u}{\partial t}+\rho u\cdot \nabla =\nabla \left[-p+\left(\mu +\mu_{T}\right)\left(\nabla u+{\left(\nabla u\right)}^{T}\right)\right]-\nabla \cdot \left\{\left[\rho C_{d}\left(1-C_{d}\right)\right]u_{slide}u_{slide}\right\}+F,$$

where:

*C_d_*—mass density of dispersed phase,
***F***—additional volumetric forces,
*p*—pressure,
*t*—time,
***u***—velocity,
***u****_slide_*—velocity in-between phases,
*μ*—dynamic viscosity,
*μ_T_*—turbulent viscosity,
*ρ*—density;

and:

(2) $$\rho =\varphi_{c}\rho_{c}+\varphi_{d}\rho_{d},$$

where:

*ρ_c_*—density of continuous phase,
*ρ_d_*—density of dispersed phase,
*φ_c_*—volumetric share of continuous phase,
*φ_d_*—volumetric share of dispersed phase;

and:

(3) $$C_{d}=\frac{\varphi_{d}\rho_{d}}{\rho }.$$

For a continuous fluid (in this case, it is air), viscosity is constant. The left side of the equation represents a temporal variation in momentum and gas acceleration. The right side represents the pressure gradient force (the normal stress) and the viscous force (the tangential shear stress). The equation of continuity (4) for the mixture of the dispersed fluid (oil) and continuous phase (air) is obtained by applying the principle of mass conservation to a small volume of fluid. The standard (general) form for Cartesian coordinates is as follows [57]:

(4) $$\frac{\partial \rho }{\partial t}+\nabla \cdot \left(\rho u\right)=0.$$

The flow of a dispersed phase is represented by the following Equation (5):

(5) $$\frac{\partial \varphi_{d}}{\partial t}+\nabla \cdot \varphi_{d}\left[u+\varphi_{d}\left(1-C_{d}\right)u_{slide}-\frac{D_{md}}{\varphi_{d}}\nabla \varphi_{d}\right]=-\frac{m_{dc}}{\rho_{d}},$$

where:

*D_md_*—diffusion coefficient of dispersed phase in a fluid,
*m_dc_*—mass transfer between phases.

Because a fluid-gas drag function is used, the Schiller–Neumann model was applied. It is described by the following Equation (6):

(6) $$\frac{3}{4}\frac{f_{d}}{d_{d}}\rho_{c}\left|u_{slide}\right|u_{slide}=\frac{\rho -\rho_{d}}{\rho }\nabla p,$$

where:

*d_d_*—diameter of dispersed phase,
*f_d_*—coefficient of flow resistance for dispersed phase.

The correct limiting behaviour in the inertial regimes is ensured by the limitation of Reynolds number as follows (7):

(7) $$f_{d}=\left\{\begin{array}{c} \frac{24}{Re_{p}}\left(1+0.15Re_{p}^{0.687}\right)\\ 0.44 \end{array}for\;\begin{array}{c} Re_{p}\\ Re_{p} \end{array}\begin{array}{c} <1000\\ >1000 \end{array}\right\},$$

where Reynolds number is described by the following equation:

(8) $$Re_{p}=\frac{d_{d}\rho_{c}\left|u_{slide}\right|}{\mu }.$$

The heat exchange is described by total energy equation as follows:

(9) $$\frac{\partial (\rho h_{tot})}{\partial t}-\frac{\partial p}{\partial t}+\nabla \left(\rho Uh_{tot}\right)=\nabla \left(\lambda \nabla T\right)+S_{E},$$

where $h_{tot}$—the total enthalpy is described by equation:

(10) $$h_{tot}=h+\frac{1}{2}U^{2}.$$

An SST model was used for turbulence. This two-equation model is the simplest and the most popular choice for modelling turbulence for similar cases. This is because of the fact that two different transport equations characterise two independent properties of the turbulent flow. Moreover, it is robust, economical, and reasonably accurate for a large variety of turbulent flows [58,59].

Turbulence kinetic energy is described by Equation (11):

(11) $$\rho \frac{\partial k}{\partial t}+\rho u\cdot \nabla k=\nabla \cdot \left[\left(\mu \sigma_{k}\mu_{T}\right)\nabla k\right]+P_{k}-\rho \beta^{*}k\omega ,$$

where:

*k*—kinetic energy,
*β*, *σ_k_*—model constants;

and:

(12) $$P_{k}=\mu_{T}\left(\nabla u:\left(\nabla u+{\left(\nabla u\right)}^{T}\right)-\frac{2}{3}{\left(\nabla \cdot u\right)}^{2}\right)-\frac{2}{3}\rho k\nabla \cdot u,$$

and:

(13) $$\mu_{T}=\rho \frac{k}{\omega }.$$

The grade of the dissipation of the energy is described by Equation (14):

(14) $$\rho \frac{\partial \omega }{\partial t}+\rho u\cdot \nabla \omega =\nabla \cdot \left[\left(\mu +\sigma_{\omega }\mu_{T}\right)\nabla \omega \right]+\varphi \frac{\omega }{k}P_{k}-\rho \beta \omega^{2},$$

where:

*P_k_*—turbulence production,
*σ_ω_*—model constant,
*ω*—turbulent frequency.

The SST model makes it possible to avoid miscalculations of the turbulence by the wall. At minimum values, the Reynolds number is switched over to the wall function. The SST model correctly calculates the turbulence in such cases as sudden stream expansions (like at the end of wheel channel).

### 3.3. The Results of Simulation Tests and Their Analysis

The results obtained for 27 variants of the simulations carried out in accordance with the experimental plan made it possible to determine the MMRO together with the values of the multidimensional correlation coefficient *R*, which is a measure of model adequacy assessment. It was assumed that MMRO would be described as a first-degree exponential function without interaction. A total of 18 mathematical models of the research object were determined, six for each simulation variant, differing in the value of peripheral wheel speed v_s_. The models developed describe the effect of change in the number of channels in the grinding arbor *n_channels_* and change in the angle *α* of inclination of the CCA delivery line outlets on the flow velocity of compressed cooled air *v_CCA_*, air-oil aerosol flow velocity *v_MQL_*, maximum *Θ_GWAS max_* and minimum grinding wheel temperature *Θ_GWAS min_* and maximum *Θ_w max_* and minimum workpiece temperature *Θ_w min_* in the system under analysis.

**Figure 4:**
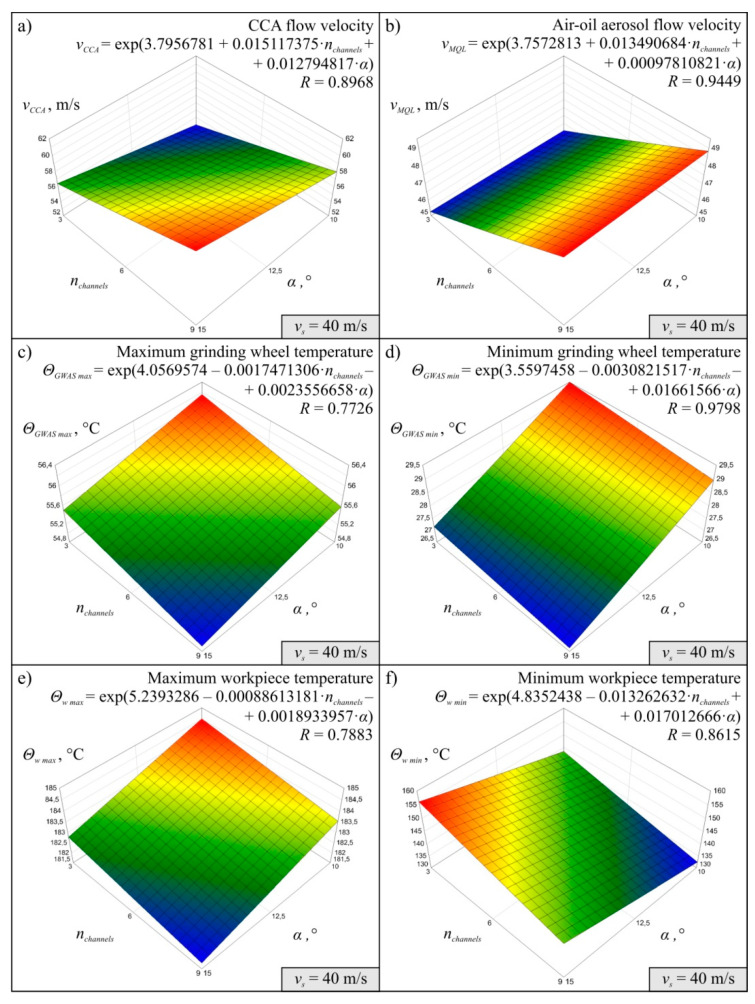
Mathematical models determined on the basis of simulation results for *v_s_* = 40 m/s: (**a**) *v_CCA_*; (**b**) *v_MQL_*; (**c**) *Θ_GWAS max_*; (**d**) *Θ_GWAS min_*; (**e**) *Θ_w max_*, (**f**) *Θ_w min._*

**Figure 5:**
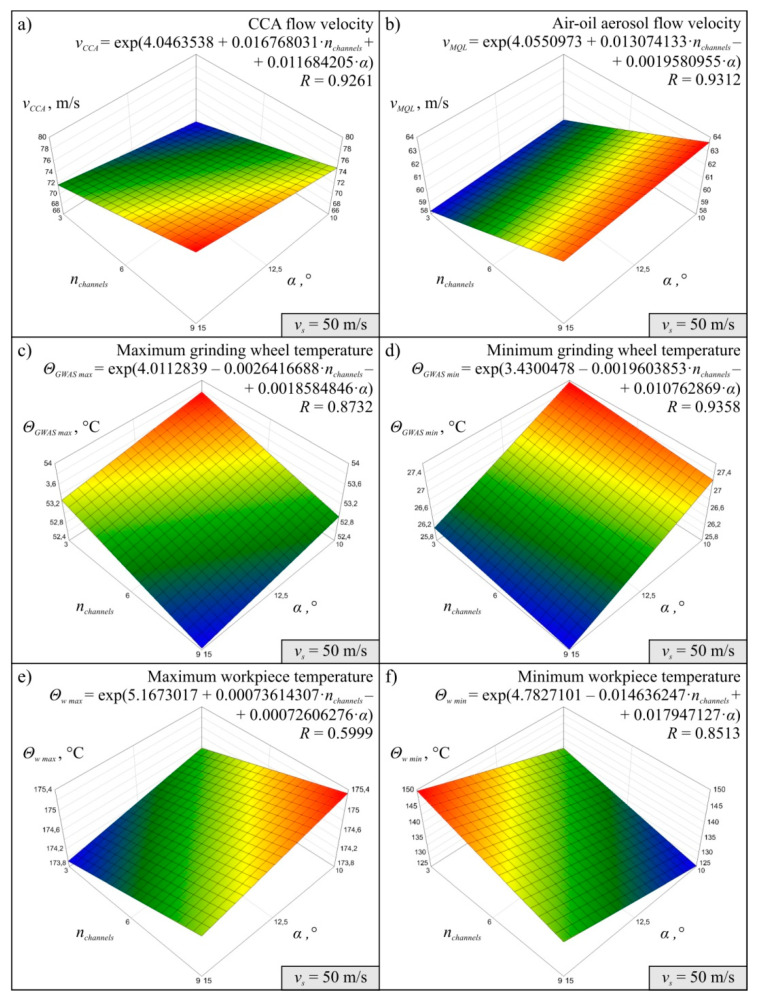
Mathematical models determined on the basis of simulation results for *v_s_* = 50 m/s: (**a**) *v_CCA_*; (**b**) *v_MQL_*; (**c**) *Θ_GWAS max_*; (**d**) *Θ_GWAS min_*; (**e**) *Θ_w max_*, (**f**) *Θ_w min._*

**Figure 6:**
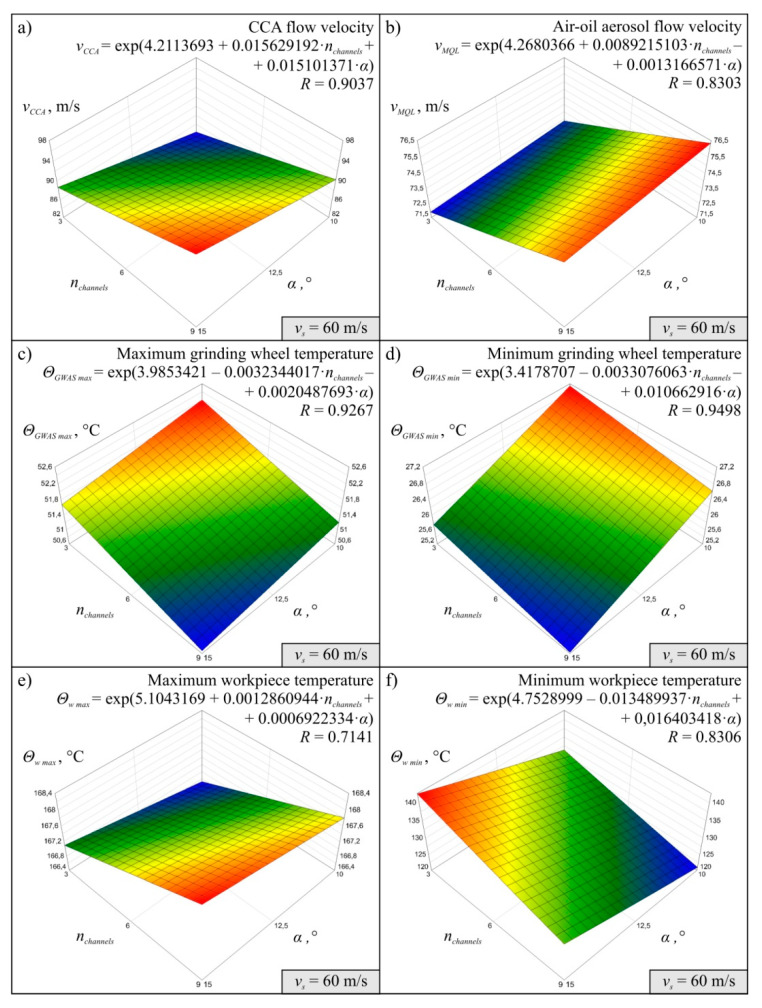
Mathematical models determined on the basis of simulation results for *v_s_* = 60 m/s: (**a**) *v_CCA_*; (**b**) *v_MQL_*; (**c**) *Θ_GWAS max_*; (**d**) *Θ_GWAS min_*; (**e**) *Θ_w max_*, (**f**) *Θ_w min._*

**Figure 7:**
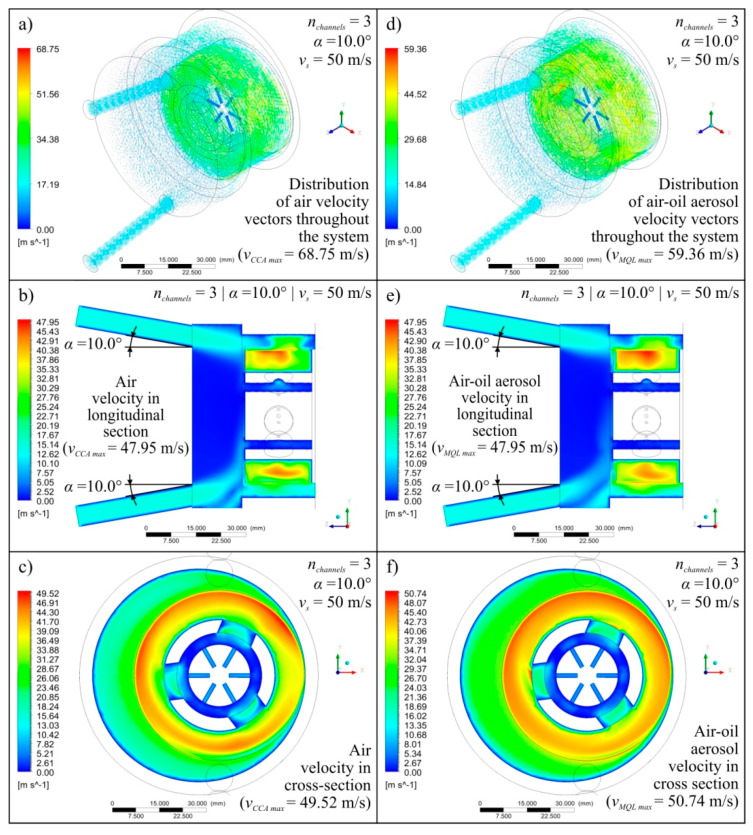
Exemplary simulation results for *α* = 10.0°, *n_channels_* = 3 and *v_s_* = 50 m/s presented as colour maps of air flow velocity (**a**–**c**) as well as oil mist flow velocity (**d**–**f**) in the entire system (**a**,**d**), in longitudinal section (**b**,**e**) and in cross-section (**c**,**f**).

**Figure 8:**
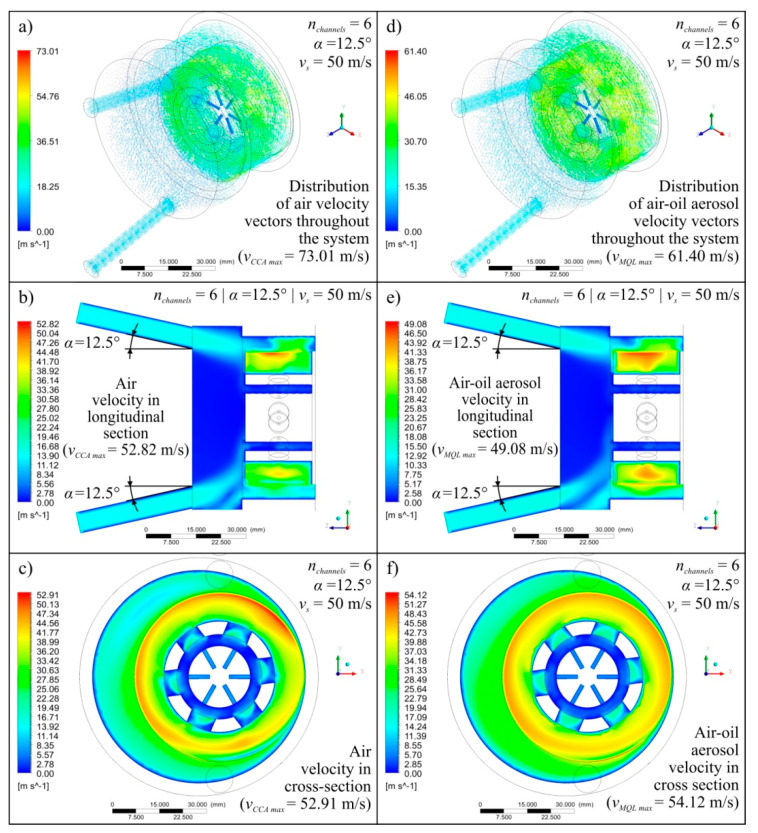
Exemplary simulation results for *α* = 12.5°, *n_channels_* = 6 and *v_s_* = 50 m/s presented as colour maps of air flow velocity (**a**–**c**) as well as oil mist flow velocity (**d**–**f**) in the entire system (**a**,**d**), in longitudinal section (**b**,**e**) and in cross-section (**c**,**f**).

**Figure 9:**
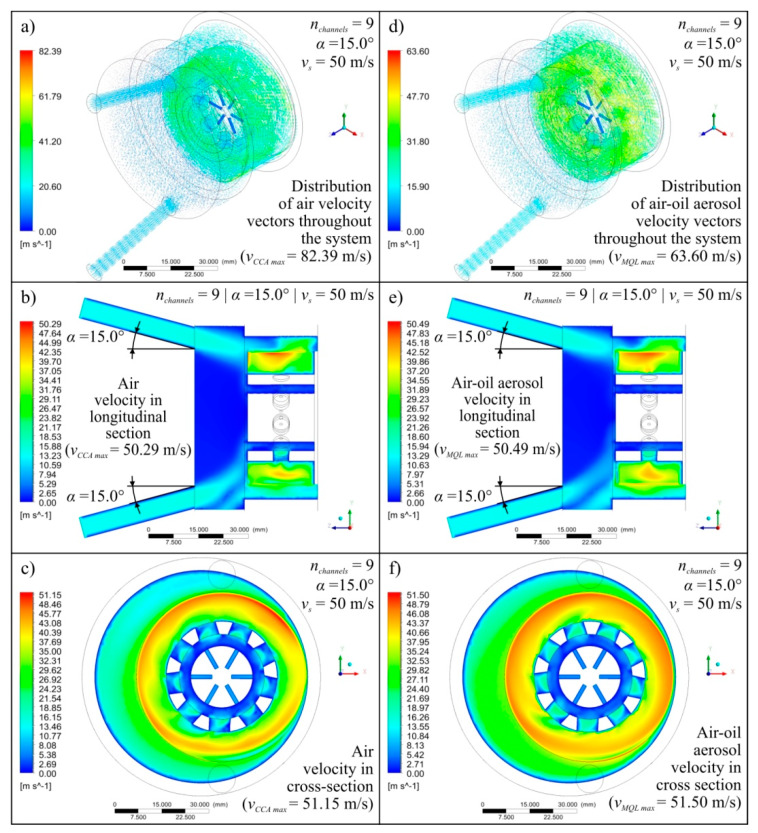
Exemplary simulation results for *α* = 15.0°, *n_channels_* = 9 and *v_s_* = 50 m/s presented as colour maps of air flow velocity (**a**–**c**) as well as oil mist flow velocity (**d**–**f**) in the entire system (**a**,**d**), in longitudinal section (**b**,**e**) and in cross-section (**c**,**f**).

**Figure 10:**
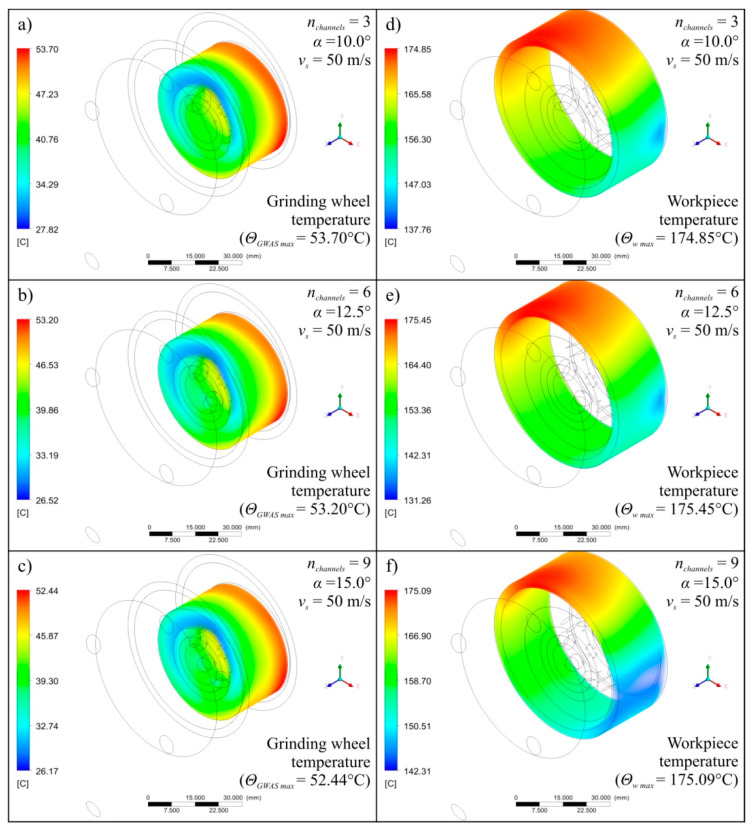
Exemplary simulation results presented as colour maps of grinding wheel temperature (**a**–**c**) as well as oil mist flow velocity (**d**–**f**) for *α* = 10.0°, *n_channels_* = 3, *v_s_* = 50 m/s (**a**,**d**), for *α* = 12.5°, *n_channels_* = 6, *v_s_* = 50 m/s (**b**,**e**) and for *α* = 15.0°, *n_channels_* = 9, *v_s_* = 50 m/s (**c**,**f**).

Diagrams showing the variability of the determined mathematical models together with their equations and values of *R* coeficient were shown in Figure 4 (for *v_s_* = 40 m/s), Figure 5 (for *v_s_* = 50 m/s), and Figure 6 (for *v_s_* = 60 m/s). The following figures show selected detailed simulation results in the form of colour maps of air velocity and air-oil aerosol distribution in the whole system, in longitudinal and cross-sectional sections (Figure 7, Figure 8 and Figure 9), as well as the distribution of grinding wheel and workpiece temperature (Figure 10). These figures present simulation results for three selected variants corresponding to the following combinations of input parameters:

- *α* = 10.0°, *n_channels_* = 3 and *v_s_* = 50 m/s—Figure 7, Figure 10a,d;
- *α* = 12.5°, *n_channels_* = 6 and *v_s_* = 50 m/s—Figure 8, Figure 10b,e;
- *α* = 15.0°, *n_channels_* = 9 and *v_s_* = 50 m/s—Figure 9, Figure 10c,f.

The simulation results proved that both the CCA and air-oil aerosol flow velocity increases as the number of channels in the grinding arbor through which the coolant generated by the MQL nozzle is delivery increases (Figure 4, Figure 5 and Figure 6). The velocity *v_CCA_* also increases with increasing the outlet angle value *α* within the range of its variability tested (Figure 4a, Figure 5a and Figure 6a). However, the angle *α* does not have a clear effect on the speed value of *v_MQL_* describing the movement of the air-oil aerosol delivered by the MQL nozzle centrifugally through the grinding arbor and the grinding wheel to the grinding zone (Figure 4b, Figure 5b and Figure 6b). As the speed of the cooling lubricants (in this case CCA and air-oil aerosol) increases, heat is taken away from the solids (grinding wheel and workpiece) by the flowing gas (air) and liquid (oil). This phenomenon is visible on the diagrams presenting values of *Θ_GWAS max_* (Figure 4c, Figure 5c and Figure 6c), *Θ_GWAS min_* (Figure 4d, Figure 5d and Figure 6d) and *Θ_w min_* (Figure 4f, Figure 5f and Figure 6f). It should be noted that the greatest influence of *n_channels_* and *α* parameters was obtained for minimum values of the workpiece temperature *Θ_w min_* (Figure 4f, Figure 5f and Figure 6f). The values of the maximum workpiece temperature *Θ_w max_* (Figure 4e, Figure 5e and Figure 6e) changed least significantly as a function of these parameters (*n_channels_* and *α*). Such small variability of this resultant factor caused that difficulties with determination of MMRO of high adequacy were encountered and the calculated multidimensional correlation coefficient *R* assumed relatively small values for these cases: *R* = 0.7883 (Figure 4e), *R* = 0.5999 (Figure 5e) and *R* = 0.7141 (Figure 6e). As a result, the slope of the surface resulting from the regression equation *Θ_w max_* is different for subsequent variants corresponding to the three values of *v_s_* adopted in the study (Figure 4e, Figure 5e and Figure 6e), which does not give the possibility of unambiguous conclusion about the influence of input factors on the analysed parameter. In the case of the remaining MMROs, the values of the *R* coefficient ranged from *R* = 0.8303 (Figure 6b) to *R* = 0.9798 (Figure 4d). Taking into account the stochastic nature of the grinding process, which is characterised by a large number of variables and many interferences, the obtained values of the *R* coefficient should be considered satisfactory and prove the acceptable adequacy of the models determined.

A more in-depth analysis of the causes of the revealed dependencies was possible based on the results of 27 detailed points of the experimental plan. Figure 7, Figure 8, Figure 9 and Figure 10 show the most important of the analysed simulation results for selected three combinations of input factor values (*α*, *n_channels_* and *v_s_*). Colour maps of changes in velocity of *v_CCA_* and *v_MQL_* and distribution of their vectors (Figure 7, Figure 8 and Figure 9), as well as maps of temperature distribution of grinding wheel and workpiece (Figure 10), make it possible to trace the path taken by CCA and air-oil aerosol from their delivery points to the grinding zone. The asymmetry in the grinding wheel and workpiece temperature distribution resulting from the direction of the main coolant agent (CCA) delivery in the analysed process is also visible.

The above described trends of change are the same for all three variants of the peripheral speed of grinding wheel applied in simulation tests. Their results showed the expected increase in the flow velocity of the cooling and lubrication media together with the increase in value v_s_. The simulation tests therefore show that the most favourable conditions (taking into account heat exchange) can be obtained with grinding at a speed of *v_s_* = 60 m/s (the highest in the assumed range of variability of this parameter).

The obtained results of the simulation tests made it possible to select the following parameters of the system under consideration as the most favourable ones:

- number of channels in grinding arbor for air-oil aerosol centrifugal delivery: *n_channels_* = 9;
- the diameter of a single channel in the grinding arbor for air-oil aerosol centrifugal delivery: *d_channels_* = 5.00 mm;
- alignment angle in the *z* axis of the CCA supply line outlets: *α*_1_ = *α*_2_ = 15.0°;
- peripheral speed of grinding wheel: *v_s_* = 60 m/s;
- peripheral speed of the workpiece: *v_w_* = 1.00 m/s.

## 4. Conclusions

The results of the numerical simulations described in this article prove that it is possible to achieve a beneficial effect of combining the centrifugal MQL method and CAG nozzle cooling in the internal cylindrical grinding process. Analyses carried out in relation to many criteria of the grinding process evaluation allowed the following conclusions to be drawn.

1. Simulation studies showed that both the number of channels in the grinding arbor *n_channels_* and the angle of inclination of the CAG nozzle outlets *α* have a significant impact on the coolant flow rate and thermal conditions in the grinding zone.
2. The results of the simulation tests indicate that a larger number of channels in the grinding arbor (from the range under test) has the effect of increasing the maximum coolant flow velocity (air flow velocity and oil flow velocity) in the grinding zone due to a local increase in the oil and air particle flow velocity in the volume of the grinding arbor channels, which was caused by the smaller value of the cross-sectional area of a single channel through which the coolant flowed.
3. Variants of the system with nine channels in the grinding arbor allowed for a noticeable reduction in the temperature of the workpiece, which resulted from a more even distribution of the air-oil aerosol on the perimeter of the inner cylindrical surface of the workpiece domain, in relation to variants with six and three channels in the grinding arbor.
4. The 15° angle of inclination of the CAG supply line outlets affects the concentration of the greater part of the high-speed CCA jet on the surface of the grinding wheel, which enables increased cooling efficiency. The 10° inclination of the CAG supply line outlets affects the concentration of the greater part of the CCA jet on the workpiece surface, which enables an increased efficiency of workpiece cooling.
5. As the peripheral speed of the grinding wheel domain increased (in the examined range), the maximum flow speed of the coolant in the grinding zone and the cooling efficiency of both the grinding wheel domain and the workpiece increased.
6. The lower speed of the air-oil aerosol flow through the intergranular spaces translates into less effective heat removal from the grinding wheel body.
7. The results obtained for the simulation tests made it possible to visualise the velocity vectors of the two-phase coolant flow in a complex system of centrifugal air-oil aerosol delivery through an open structure of a very fast rotating porous layer (grinding wheel), with an additional CCA supply using an external CAG nozzle, which is an important cognitive aspect and allows for further development of research in this field.
8. The simulation tests carried out enabled a precise selection of the conditions for delivery the coolant to the grinding zone for further experimental testing of the analysed internal cylindrical grinding process. The results of experimental research are described in detail in the paper [60]. They confirm that with the application of the most advantageous process conditions resulting from the simulation tests it is possible to achieve effective penetration of cooling and lubricating agents into the grinding zone. Moreover, it was proved that directing the CCA stream directly behind the grinding wheel’s contact zone with the machined material effectively cleans the working area from chips, thus limiting the phenomenon of clogging of the GWAS.

## Figures and Tables

**Table 1:** Selected physical and mechanical properties of 100Cr6 steel.

Group of Prosperities	Property	Unit	Value
Physical	Poisson number	*v*	0.30 ^(2)^
	Specific heat	J/kg·K	475 ^(1)^
	Density	kg/dm^3^	7.81
	Thermal conductivity	W/m·K	46.6
	Electrical resistivity	Ω·mm^2^/m	0.22
	Electrical conductivity	S·mm^2^/m	4.55
Mechanical	Thermal expansion	10^−6^·1/K	11.4 ^(1)^
	Modulus of elasticity	longitudinal GPa	210 ^(2)^
		tangential GPa	80 ^(2)^
	Bulk modulus	GPa	140 ^(2)^
	Poisson number	*v*	0.30 ^(2)^

^(1)^ Value calculated for the temperature 20–100 °C; ^(2)^ Value calculated for the temperature of 20 °C.

**Table 2:** Assumptions of simulation.

**Assumptions**	Total channel cross-sectional area in the grinding arbor *A_channels_* = 176.7 mm^2^ for each of the geometrical variants under consideration
	Grinding wheel offset from the workpiece surface by 0.2 mm
	The GWAS and the face of the grinding wheel are rough (*R_SGR_* = 0.425 mm)
	Only the main rotational movement of the grinding wheel and the basic feed movement (counter-rotation) of the workpiece are taken into account
	No impact of the environment on airflow and flow of air-oil aerosol
	The air has the properties of an excellent gas
	The air and air-oil aerosol mass is relatively low
	The heat exchange takes place through forced convection and heat conduction
	On the basis of exploratory thermovision measurements it is assumed that the heat source is the external surface of the grinding wheel (*T_GWAS_* = 80 °C) and the internal surface of the workpiece (*T_w_* = 180 °C)
	The walls of the CCA supply line outlets and MQL nozzle outlets are insulated
	The grinding wheel material is Al_2_O_3_ with a porous structure
	Values *v_s_* were changed at the same time from *v_w_* with a constant value of the ratio *q* = *v_s_*/*v_w_* = 60

**Table 3:** Simplifications of simulation.

**Simplifications**	The number of geometric simulation variants has been limited
	Friction and contact issues between the GWAS and the workpiece surface are omitted
	All other walls are modelled as smooth
	Axial wheel feed *v_fa_* and radial wheel feed *v_fr_* have been omitted in order to simplify the system kinematics and shorten the time of simulation calculations
	Only the volume of the environment in the form of unhindered air is taken into account
	The intermolecular interactions are reduced only to repulsion at the moment of perfectly resilient collisions. The particles are in constant chaotic motion and their volume is negligible in relation to the gas volume.
	The effect of earth acceleration on the CCA flow in the system under consideration shall be ignored
	The contribution of radiation to the heat exchange case under consideration is ignored
	The influence of other possible heat sources, such as machine elements or room light sources, shall be ignored
	The heat exchange between the environment and the walls of the CCA supply line as well as MQL nozzle is ignored
	The share of the grinding wheel bond in the simulation is omitted

**Table 4:** Characteristics of simulation model.

**Geometric Parameters of the Model**	Outer diameter of grinding wheel: *d_s_* = 40.0 mm			
	Internal diameter of the workpiece: *d_w_* = 50.0 mm			
	Number of channels in the grinding arbor: *n_channels_* = 3, 6, 9			
	Diameter of a single channel in the grinding arbor for air-oil aerosol delivery: *d_channels_* = 5.00 mm; 6.12 mm; 8.66 mm			
	CCA supply line diameter: 6.3 mm			
	Angle of the CCA supply line outlet: *α*_1_ = *α*_2_ = 10.0°; 12.5°; 15.0°			
**Discretisation Mesh Parameters of the Model**	*n_channels_* = 3	*α*_1_ = *α*_2_ = 10.0°	334972 elements	92769 nodes
	*n_channels_* = 3	*α*_1_ = *α*_2_ = 12.5°	332469 elements	91645 nodes
	*n_channels_* = 3	*α*_1_ = *α*_2_ = 15.0°	331659 elements	91361 nodes
	*n_channels_* = 6	*α*_1_ = *α*_2_ = 10.0°	355312 elements	97239 nodes
	*n_channels_* = 6	*α*_1_ = *α*_2_ = 12.5°	349753 elements	95381 nodes
	*n_channels_* = 6	*α*_1_ = *α*_2_ = 15.0°	349561 elements	95526 nodes
	*n_channels_* = 9	*α*_1_ = *α*_2_ = 10.0°	367551 elements	100119 nodes
	*n_channels_* = 9	*α*_1_ = *α*_2_ = 12.5°	363216 elements	98483 nodes
	*n_channels_* = 9	*α*_1_ = *α*_2_ = 15.0°	362811 elements	98615 nodes
	mesch	domain: fluid	min. size 0.1 mm	max. size 2 mm
**Properties of Liquids and Solids Used in the Simulation**	Air	Model: ideal gas from the ANSYS^®^ library		
		Morphology: fluid in continuous phase		
	Oil	Model: defined Cimtech MQL oil model from Cimcool		
		Morphology: dispersed fluid (droplet size 0.002 mm)		
	Steel (workpiece)	Model: default steel model from the ANSYS^®^ library		
		Morphology: solid in continuous phase		
	Al_2_O_3_ (grinding wheel)	Model: defined model: Al_2_O_3_		
		Morphology: solid in continuous phase		

**Table 5:** Conditions of simulation process.

**Workstation**	Dell Precision T7500 Workstation (Intel Xeon X5690 3.47 GHz, 24 GB RAM)	
**Operating System**	Windows 7 Professional 64 bit	
**Software**	ANSYS^®^ 18.1	
**Conditions of the Simulation Process**	Type of analysis: steady state	
	Time step for fluids: physical time step: 3 ×·10^−5^ s	
	Time step for solids: automatic	
	Computational time: 1.409 × 10^4^ s (average)	
	Multi-phase model: two heterogeneous phases	
	Heat exchange model: total energy model	
	Continuous phase turbulence model: shear stress transport model	
	Dispersed phase turbulence model: model based on the dispersed phase zero equation	
	Reference pressure value: 101,325 Pa	
	Gravity influence model: no gravity influence	
	Degree of turbulence intensity: average (intensity 5%)	
	Flow condition: subsonic	
	Total volumetric flow rate of CCA: *Q_CCA_* = 0.00083 dm^3^/s	
	CCA feed rate: *v_CCA_* = 13.3 m/s	
	Grinding wheel peripheral speed: *v_s_* = 40 m/s, 50 m/s, 60 m/s	
	Workpiece peripheral speed: *v_w_* = 0.67 m/s, 0.83 m/s, 1.00 m/s	
	Ambient air start temperature: 18 °C	
	Supply CCA temperature: –5 °C	
	Grining wheel (Al_2_O_3_)	Heat transfer coefficient: 300 W/m^2^·K Outer limit temperature: 18 °C Heat source: flux of 55,000 W/m^2^ Thermal conductivity: 38.5 W/m·K Volumetric heat capacity: 39.1 × 10^2^ J/dm^3^·K
	Workpiece (steel)	Heat transfer coefficient: 100 W/m^2^·K Outer limit temperature: 18°C Heat source: flux of 45,000 W/m^2^ Thermal conductivity: 60.5 W/m·K Volumetric heat capacity: 33.9 × 10^2^ J/dm^3^·K

## Nomenclature

*Symbols*	
*a_e_*	working engagement (machining allowance), mm
*A_channels_*	summary cross-sectional area of the channels in the grinding arbor, mm^2^
*C_d_*	mass density of dispersed phase, kg/m
*d_channels_*	grinding arbor channels diameter, mm
*d_s_*	grinding wheel outer diameter, mm
*d_w_*	workpiece internal diameter, mm
*D_md_*	diffusion coefficient of dispersed phase in a fluid, m/s^2^
***F***	additional volumetric forces, N/kg
*f_d_*	coefficient of flow resistance for dispersed phase
*G*	grinding ratio
*m_dc_*	mass transfer between phases
*n_channels_*	number of channels in the grinding arbor
*n_s_*	grinding wheel rotational speed, rpm
*n_w_*	workpiece rotational speed, rpm
*p*	pressure, Pa
*P_k_*	turbulence production
*q*	speed ratio *q* = *v_s_*/*v_w_*
*Q_CCA_*	compressed cold air flow rate, dm^3^/min
*R*	multidimensional correlation coefficient
*R_GWAS_*	roughnes of the grinding wheel active surface
*t*	time, s
*T*	temperature, °C
*T_GWAS_*	temperature of the heat source on the external surface of the grinding wheel, °C
*T_w_*	temperature of the heat source on the internal surface of the workpiece, °C
***u***	velocity, m/s
*U*	work, J
***u*** *_slide_*	velocity in-between phases, m/s
*u_τ_*	friction velocity, m/s
*v_c_*	volume of continuous phase, m^3^
*v_CCA_*	compressed cooled air flow rate, m/s
*v_d_*	volume of dispersed phase, m^3^
*v_fo_*	axial feed velocity of the grinding wheel, mm/s
*v_fr_*	radial feed velocity of the grinding wheel, mm/s
*v_MQL_*	air-oil aerosol flow rate, m/s
*v_s_*	grinding wheel peripheral speed, mm/s
*v_w_*	workpiece peripheral speed, mm/s
*Greek symbols*	
*α*_1_	alignment angle of compressed cooled air supply line outlet no. 1, °
*α*_2_	alignment angle of compressed cooled air supply line outlet no. 2, °
*β*	model constant
*ε*	dissipation rate of turbulent energy
*Θ_GWAS max_*	maximum grinding wheel temperature, °C
*Θ_GWAS min_*	minimum grinding wheel temperature, °C
*Θ_w max_*	maximum workpiece temperature, °C
*Θ_w min_*	minimum workpiece temperature, °C
*μ*	dynamic viscosity, Pa·s
*μ_T_*	turbulent viscosity, Pa·s
*ν*	kinematic viscosity, m^2^/s
*ρ*	density, kg/m^3^
*ρ_c_*	density of continuous phase, kg/m^3^
*ρ_d_*	density of dispersed phase, kg/m^3^
*σ_k_*	model constant
*σ_ω_*	model constant
*φ_c_*	volumetric share of continuous phase
*φ_d_*	volumetric share of dispersed phase
*ω*	turbulent frequency
*Acronyms*	
CAG	Cold Air Gun
CAMQL	Cold Air Minimum Quantity Lubrication
CAOM	Cold Air and Oil Mist
CCA	Compressed Cooled Air
CFD	Computational Fluid Dynamics
GWAS	Grinding Wheel Active Surface
MMRO	Mathematical Model of the Research Object
MQC	Minimum Quantity Cooling
MQCL	Minimum Quantity Cooling Lubrication
MQL	Minimum Quantity Lubrication
